# Optimization of *Eugenia punicifolia* (Kunth) D. C. leaf extraction using a simplex centroid design focused on extracting phenolics with antioxidant and antiproliferative activities

**DOI:** 10.1186/s13065-020-00686-2

**Published:** 2020-04-27

**Authors:** Catarina dos Santos, Andressa Lie Mizobucchi, Bruna Escaramboni, Bruno Pereira Lopes, Celio Fernando Figueiredo Angolini, Marcos Nogueira Eberlin, Karina Alves de Toledo, Eutimio Gustavo Fernández Núñez

**Affiliations:** 1 Laboratory of Chemistry of UNESP-Assis (LAQUA), Department of Biological Sciences, Faculty of Sciences and Letters, University of São Paulo State (UNESP), Sto Antonio Ave, 19806-900 Assis, SP Brazil; 2 Department of Biotechnology, Faculty of Sciences and Letters, University of São Paulo State (UNESP), 19806-900 Assis, SP Brazil; 3 Laboratory of Physiopathology of Innate Immunity, Department of Biological Sciences, Faculty of Sciences and Letters, University of São Paulo State (UNESP), 19806-900 Assis, SP Brazil; 4 Mass Spectrometry and Chemical Ecology Laboratory (MS-Cell), Center for Natural and Human Sciences, Federal University of ABC (UFABC), Santo André, São Paulo, SP Brazil; 5 Thomson Mass Spectrometry Laboratory, Institute of Chemistry, University of Campinas (UNICAMP), Campinas, São Paulo, Brazil; 6 Mackenzie Presbyterian University, Mackenzie Research Nucleus in Science, Faith and Society, Maria Antônia Street, 163 Room 44, Vila Buarque, 01222010 São Paulo, SP Brazil; 7 School of Arts, Sciences and Humanities, University of São Paulo (EACH-USP), Arlindo Béttio Street, 1000 - Vila Guaraciaba, 03828-000 São Paulo, SP Brazil

**Keywords:** Mixture design, *Eugenia punicifolia*, Extraction, Antioxidant, Antiproliferative activities

## Abstract

*Eugenia punicifolia* (Kunth) D. C. (Myrtaceae) has been showing interesting biological activities in the literature which was correlated to its phenolic compounds. In the sense of a better recovering of phenolics with the best antioxidant and antiproliferative activities, an extraction, based on multivariate analytical approach, was developed from *E. punicifolia* leaves. The different extractor solvents (ethanol, methanol and water) and their binary and ternary combinations were evaluated using a simplex-centroid mixture design and surface response methodology. The optimized crude extracts were investigated for phenol and flavonoid content and compared to their antioxidant (EC_50_) and antiproliferative properties against HEp-2 (cell line derived from the oropharyngeal carcinoma) and mononuclear viability cells. Ethanolic extracts showed the best phenolic content with the highest antioxidant activity and moderated activity antiproliferative to HEp-2. ESI-QTOF–MS revealed the presence of quercetin and myricetin derivatives, which was correlated to activities tested. Then, simplex-centroid design allowed us to correlate the *Eugenia punicifolia* biological activities with the extracts obtained from solvent different polarity mixtures.
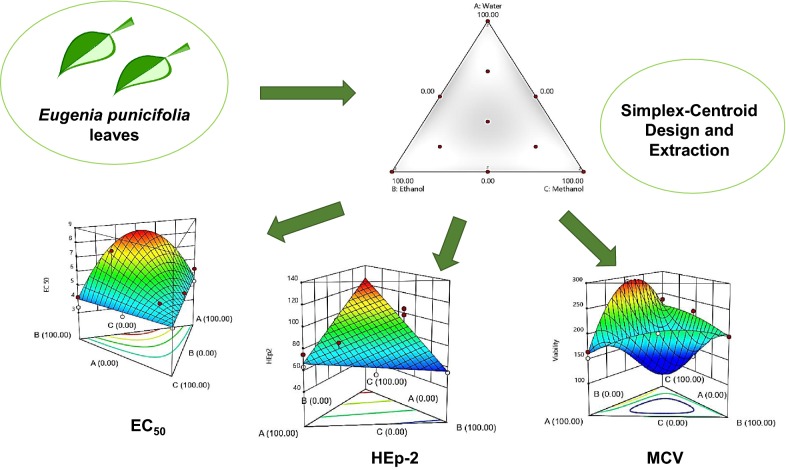

## Introduction

Many biological activities are related to the phenolics compounds. They are only not synthesized in plants for growth or reproduction, but to perform genetic, physiological, or biochemical roles [1, 2]. This class of compounds contributes to plant adaptation within its environment through activities like UV protection and defence against pathogens, animals and other plants. Which has led to their use for obtaining high value-added products like fine chemicals, phytotherapics, cosmetics and nutraceutical compounds [3]. Besides, these phytochemicals have been extensively described in the literature and traditional medicine.

Some of these phenolic compounds are antioxidants of natural origin receive increasing consumer preference over those of synthetic origin, due to factors such as tolerance, safety, a very low toxicity and the absence of side effects [4]. Antioxidants inhibit the initiation and propagation of oxidative chain reactions, which are responsible for generating reactive oxygen species (ROS), thus preventing the development of degenerative diseases and cancer [5]. Some recent evidence shows phenolics directly interact with proteins, making them ideal small molecules for modulating enzymes, transcription factors and receptors.

These phenolics are widely found in the *Eugenia* spp. (Myrtaceae) leaf extracts [6, 7] and fruits [8]. Myrtaceae are well known for their economic importance since they are cultivated worldwide. Guava (*Psidium*), jaboticaba (Myrciaria), pitanga (Eugenia) trees has commercial application and biological activity studies [9, 10]. From *Eugenia punicifoli*a (Kunth) D. C. leaves it have reported some pharmacological activities like the recovery of inhibitory effects in nicotinic-cholinergic neurotransmission by its aqueous extract in rat diaphragm [11]; antioxidant activity and inhibited enzymes related to metabolic syndrome [12]; antinociceptive, anti-inflammatory and gastroprotective effects in rodents [6]; inflammation inhibition and skeletal muscle remodeling activation using a polymer implant containing pentacyclic triterpenes from *E. punicifolia* [13]; and ulcer-healing [14].

Previous studies indicated *E. punicifolia* hydroethanolic leaf extracts are a rich quercetin and myricetin source [6, 7]. Quercetin aglycones are found to play a critical role in the immunomodulatory action in inflammatory responses against H_2_O_2_, leading to a decrease in the generation of ROS (reactive oxygen species) [15, 16]. Myricetin exhibits a wide range of activities that include strong antioxidant, anticancer, antidiabetic and anti-inflammatory activities. For this reason, It also used as a preserving agent to extend the shelf life of foods containing oils and fats, since this phenolic compound is attributed with an ability to protect lipids against oxidation [17].

Just as there is a high demand for antioxidant compounds there is also an intense demand for anticancer compounds. Cancer cells are characterized by uncontrolled cellular division and their ability to migrate to other parts of the body. Tumour cells creates a microenvironment that provides a nurturing condition for malignant processes development. Oxidative stress is maintained in the tumour microenvironment to sustain cancer progression [18]. So, redox regulation seems to be the key factor in regulating carcinogenesis and may be modulated by phenolic antioxidants, as commonly found in Myrtaceae [18] [19],. Moreover, chemotherapeutic drugs can cause serious side effects and drug resistance, which pose limiting factors in the long-term use of these drugs. So, there is a great demand for alternative anticancer agents with low side effects [20].

In general, plant phenolics are more soluble in organic solvents like methanol, ethanol and aqueous acetone solutions, but the diversity of phenolics present in plant tissues challenge the standardization of extraction methods. One possible way is selecting a mixture of extracting solvents by the changes in the solvent proportions within the systems (binary, ternary or even multicomponent mixtures) via experimental mixture design, like simplex-centroid. Simplex-centroid provides an economic and time-saving method, unlike methods like trial-and-error, because it takes advantage of statistical criteria in order to minimize both the model error and the number of required experiments [21, 22]. Besides, in this system, it is possible to observe synergistic/antagonistic effects which were result of different compounds extracted [23].

So, in this work, a simplex centroid mixture design was used to find the most adequate solvent mixture for the extraction of phenolic compounds with best antioxidant and antiproliferative activities from *E. punicifolia* leaves. The total phenolic and flavonoid contents, antioxidant and antiproliferative activities were used as responses to select the best extractor. Additionally, the phenolics compounds were identified by elestrospray ionization, in negative mode, coupled to a hybrid mass spectrometer with quadrupole and time-of-flight analyses [ESI(−)Q/TOFMS].

## Experimental section

### Drugs and chemicals

Gallic acid, quercetin, and 2,2-diphenyl-1-picrylhydrazyl (DPPH) were purchased from Sigma-Aldrich (St. Louis, MO, USA). Other analytical grade reagents were purchased locally.

### Collection and preparation of plant material

*Eugenia punicifolia* leaves were collected on January (2010) by Dr Catarina dos Santos at the Instituto Florestal e Estações Experimentais—Floresta Estadual de Assis at 22°33′ to 22°37′ Lat. S–50°21′ to 50°24′ Long. W, Assis, State of São Paulo, Brazil. The specimen was identified by Dr Antônio C. G. Melo, and a voucher specimen (no.43.522) was deposited in the Herbarium D.Bento Pickel, São Paulo, SP, Brazil, for future reference, in compliance with legal norms and registered as Cotec 206108-005.298/2009.

### Ethics statement

The experimental procedures using human blood were approved by the local Research Ethics Committee of the Faculty of Science and Letters at the University of São Paulo State Campus Assis (Approval Number 467783151.0000.5401). We obtained written consent, suggested and approved by the Committee, from each participant before initiating any research procedures.

### Mixture design and extract preparation

**Fig. 1:**
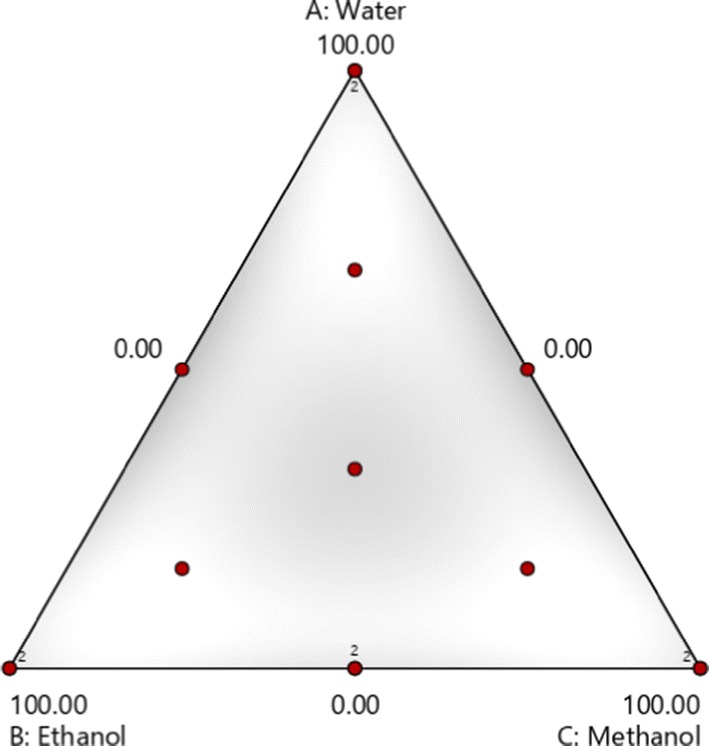
Simplex centroid design used to optimize the extraction. The points correspond to the mixtures in Table 1. The number 2 in four mixtures represents the number of experimental repetitions performed in each of them

Extraction solvents were mixtures of water, methanol and ethanol in various proportions according to a simplex centroid design, as shown in Fig. 1. The three points at the vertices of the triangle correspond to extractions carried out using pure solvents, water (w), methanol (m) or ethanol (e). The three midpoints of the sides of the triangle correspond to 1:1 binary mixtures of these solvents. Ternary mixtures using different proportions were also investigated. The solvent mixtures were obtained according to the augmented Simplex-Centroid design (Design Expert version 12 State-Ease Inc, Minneapolis, MN, USA). Each component of the mixture was studied in the proportion range from 0 to 100%, including 14 experiments, four replicates and three combinations for performing lack of fit tests for models associated with the response variables under consideration, according to the Design Expert software (Fig. 1). For each response, the most adequate model was adopted.

For extract preparation, 5 g of crushed, dry leaves were moistened with the mixture of solvents v/v (1:10 plant/solvent ratio) submitted by dynamic maceration (3 ×2 h) at room temperature (25 ± 2 °C). After filtration, these combined solutions were concentrated under reduced pressure until complete solvent elimination to provide the extracts studied on this manuscript.

### Polyphenol and flavonoid contents

The total polyphenol content was determined for all tested extracts using Folin-Ciocalteu’s assay with some modifications [24]. Thus, Folin–Ciocalteau (2.5 mL, 10% v/v) and sodium carbonate (2.0 mL, 4% w/v) solutions were added to an aliquot (0.5 ml) of ethanolic solution of each extract, followed by thorough mixing, incubation for 120 min in the dark at room temperature and absorbance measurement at 750 nm. A calibration curve (0.5–40 µg/mL) of gallic acid was used to express the results as mg gallic acid equivalent (GAE)/g dry extract. All measurements were performed in triplicate.

The total flavonoid content was determined by the aluminium chloride colorimetric assay with modifications [24]. Thus, absolute ethanol (1.5 mL), aluminium chloride (0.1 ml, 10% w/v), potassium acetate (0.1 mL, 1 M) and distilled water (2.8 mL) were added to an aliquot (0.5 mL) of ethanolic solution of each extract, followed by thorough mixing, incubation for 30 min in the dark at room temperature and measuring the absorbance at 425 nm. A calibration curve (5–60 µg/mL) of quercetin was used to express the results as mg quercetin equivalent (GAE)/g dry extract. All measurements were performed in triplicate.

### ESI(-)-Q-TOF-MS analysis

Mass spectrometry analyses were performed on an Agilent 6500 series quadrupole time-of-flight (QTOF) mass spectrometer (Agilent, Santa Clara, CA, USA), operating in the negative ion mode. The VCap voltage was maintained at 4 kV, the gas temperature was set to 290 °C, the drying gas flow rate was 11 L min^−1^, and the nebulizer pressure was set at 45 psi. The fragmentor was maintained at 175 V and the skimmer voltage was 320 V. A mixture of ions with m/z 112.9856 and m/z 966.0007 was used as internal standards. Data were recorded in a mass range of *m*/z 100–1500, with a 1 spectrum s^−1^ scan rate and a 2 spectra s^−1^ MS/MS scan rate with an isolation width of 1.3 and collision energy of 35 eV. MS/MS precursor selection was set to four precursors per cycle, where these were chosen by the abundance only mode.

### Determination of DPPH (2,2-Diphenyl-1-picrylhydrazyl) radical scavenging

The extracts for DPPH radical scavenging were estimated according to Enujiugha et al. with some modifications [25]. Thus, DPPH solution (0.5 mL, 0.03% in methanol) was added to an aliquot (2.0 mL) of methanolic solution of each extract (1.56–200 µg min^−1^), followed by vigorous mixing, incubation for 30 min in the dark at room temperature and measuring the absorbance at 517 nm. Absolute methanol (2 mL) was used as the blank (negative control) and quercetin and gallic acid solutions were used as positive controls. All tests were carried out in triplicate. The DPPH radical scavenging activity of each extract was calculated using Eq. 1:

1 $$ {\text{\%  inhibition}} = \left( {1 - \frac{\text{Abs sample}}{\text{Abs control}}} \right)x 100 $$

where Abs_sample_ is the absorbance of each extract and Abs_control_ is the absorbance of methanol in DPPH. Different sample concentrations were used to obtain antiradical curves for calculating the EC_50_ values. Antiradical curves were plotted referring to concentration on the *x*-axis and their relative scavenging capacity on the *y*-axis. The EC_50_ values were processed using Microsoft Excel 365 and Origin 8.6.

### Culture conditions

The cell line HEp-2 (human laryngeal) was maintained in cell culture flasks with RPMI-1640 media (Roswell Park Memorial Institute medium, Sigma-Aldrich, St. Louis, MO, USA) supplemented with 5% fetal bovine serum and incubated at 37 °C in 5% CO_2_. The culture medium was exchanged every 2 days, with the application of antibiotics and antimycotics to avoid contamination. Mononuclear leukocytes were obtained from human peripheral blood (10 mL) from healthy volunteers. The leukocytes were isolated by Histopaque discontinuous gradient 1077 and 1119 (Sigma-Aldrich, St. Louis, MO, USA), following the manufacturer’s recommendations. For viability testing, 5 × 10^4^ cells were used in a 96-well microplate. The gradient was centrifuged at 700×*g* and 25 °C for 30 min (380R, Hettich Zentrifugen, Tuttlingen, Alemanha) for cell collecting. The cells were newly centrifuged at 2000 rpm for 10 min (g-force 447,2), at 25 °C with saline solution (NaCl 0,9% w/v) and deposited on RPMI 5%. Cells count were carried out in a Neubauer chamber, plating 3 × 10^5^ cells per well, in 2% F12 DMEM medium (Dulbecco’s Modified Eagle’s medium).

### MTT assay

Cytotoxicity was evaluated by the colourimetric method of MTT (3-(4,5-dimethylthiazol-2-yl) 2,5-Diphenyl Tetrazolium bromide) (Sigma), which consists of indirectly measuring cell viability using the mitochondrial enzyme activity of living cells. Cells (5 × 10^4^ HEp-2 or 3 × 10^5^ mononuclear cells/well) seeded into 96-well culture plates were incubated with different concentrations of *Eugenia punicifolia*, for 24 h at 37 °C. Subsequently, MTT (0.5 mg/mL) was added to each well and incubated at 37 °C for 4 h. After incubation, formazan crystals were diluted by adding dimethyl sulfoxide (DMSO, Sigma) and the optical density (O.D.) of samples was measured in a spectrophotometer at 570 nm. Cells incubated with either medium or 50 μM H_2_O_2_ were used as negative and positive controls (100% viable to cell death), respectively [26].

### Optimization of *E. punicifolia* extraction using Simplex Centroid Design

Extraction solvents were mixtures of water (w), methanol (m) and ethanol (e) in according to a Simplex Centroid Design (Design Expert version 12 State-Ease Inc, Minneapolis, MN, USA), as shown on Fig. 1. Each component of the mixture was studied in the proportion range from 0 to 100%, including 14 experiments, four replicates and three combinations. The three points at the vertices of the triangle correspond to extractions carried out using pure solvents. The three midpoints of the sides of the triangle correspond to 1:1 binary mixtures of these solvents. Ternary mixtures using different proportions were also investigated. According to the Design Expert software (Fig. 1). A Scheffé special cubic model was expressed for each response function from the simplex-centroid design (Eq. 2):

2 $$ y = \mathop \sum \limits_{i = 1}^{q = 3} b_{i }^{*} x_{i} + \mathop \sum \limits_{i < j}^{q = 3} \mathop \sum \limits_{j}^{q = 3} b_{ij }^{*} x_{i  } x_{j  } + b_{123 }^{*} x_{1  } x_{2  } x_{3  } $$

where y is the estimated response, b* is the coefficient estimated by the least squares method and x_i_ is the independent variable, with 0 ≤ x_i_ ≤ 1 and ∑ xi = 1 (i.e., 100 wt%). The $b_{i }^{*}$ parameter is the linear coefficient related to pure component i, $b_{ij }^{*}$ is the quadratic coefficient of binary interaction for components i and j and $b_{123 }^{*}$ is the special cubic coefficient of ternary interaction for components 1, 2 and 3 [27].

For each response, the most adequate model was adopted. The mathematical models were subjected to analysis of variance (ANOVA) and best fitted response models were achieved by displaying statistical parameters such as: R^2^ (correlation coefficient), adjusted R^2^ (adjusted correlation coefficient), lack of fit, regression F-value and regression *p* value by using analysis of variance (ANOVA). From ANOVA analysis, the model was considered significant when (p > 0.0001), indicating the good fit of model. The F test was made sure no more than 0.05 (significant) and to the lack of fit was assured to be more 0.05 (insignificant). Contour plots of the responses were generated from adjusted models. The tests of statistical significance for the model and the parameters, as well as the lack of fit test, were performed at a 95% significance level. From Table 1, ANOVA followed a Bonferroni’s test was applied.

**Table 1:** Results of Simplex-Centroid experimental design for total phenols and flavonoids, EC_50_, HEp-2 cell line and mononuclear cells

Solvents				Response variables				
Extract	H_2_O	EtOH	MeOH	TPC mgGAc/g	TFC mgQ/g	EC_50_ μg/mL	HEp-2 (%)	MVC (%)
1	0.0	50.0	50.0	339.52 ± 0.01	130.22 ± 0.02	3.85 ± 0.01	104.46 ± 0.08	214.92 ± 3.88
2	0.0	100.0	0.0	344.12 ± 0.01	128.46 ± 0.01	3.39 ± 0.01	189.90 ± 0.33	196.41 ± 18.27
3	16.7	16.7	66.6	322.78 ± 0.01	119.69 ± 0.01	4.97 ± 0.01	151.30 ± 0.04	176.45 ± 1.45
4	100.0	0.0	0.0	285.66 ± 0.01	101.86 ± 0.01	4.41 ± 0.00	74.70 ± 0.10	151.02 ± 10.00
5	0.0	100.0	0.0	412.59 ± 0.01	132.63 ± 0.03	4.13 ± 0.00	54.60 ± 0.17	232.33 ± 17.96
6	0.0	0.0	100.0	330.33 ± 0.01	137.43 ± 0.02	7.71 ± 0.00	43.37 ± 0.19	213.67 ± 11.50
7	50.0	50.0	0.0	318.51 ± 0.02	107.39 ± 0.01	8.38 ± 0.00	54.33 ± 0.30	202.03 ± 4.24
8	100.0	0.0	0.0	268.92 ± 0.00	122.76 ± 0.01	5.34 ± 0.00	63.24 ± 0.15	163.96 ± 4.79
9	16.7	66.7	16.7	312.27 ± 0.01	118.16 ± 0.02	7.58 ± 0.01	65.74 ± 0.39	148.28 ± 5.69
10	0.0	50.0	50.0	335.75 ± 0.01	131.32 ± 0.04	7.43 ± 0.01	98.20 ± 0.10	228.28 ± 3.56
11	66.6	16.7	16.7	322.12 ± 0.02	103.22 ± 0.00	5.71 ± 0.01	80.51 ± 0.04	169.66 ± 0.68
12	33.3	33.3	33.3	299.95 ± 0.03	118.68 ± 0.03	5.47 ± 0.01	71.51 ± 0.29	204.27 ± 3.09
13	50.0	0.0	50.0	164.15 ± 0.02	122.76 ± 0.00	5.00 ± 0.00	94.47 ± 0.14	290.06 ± 13.60
14	0.0	0.0	100.0	324.25 ± 0.02	127.59 ± 0.02	4.25 ± 0.00	121.87 ± 0.14	236.67 ± 20.37

*GAc* Gallic Acid, *Q* quercetin

## Results and discussion

In the process of phenolics extraction from *E. punicifolia*, it was expected that interaction effects could occur when one mixture of the extractive system interacted with another, influencing the response variable. Moreover, the phenolics extraction was already reported to be affected by the chemical complexity of the studied plant and by the polarity of the solvent used for extraction. In general, good selectivity could be achieved when using solvents and their mixtures by applying design modelling and multivariate analysis [28]. The simplex-centroid design permitted quantification of the interaction effects of solvent mixtures by adjusting their proportions [29]. The presence of interactions among extractive solvents had important implications for the understanding the extractive system in our work.

### Optimization of sample preparation

*Eugenia punicifolia* dried and powdered leaves were extracted by using three pure solvents (water, methanol, and ethanol), or mixtures of these solvents provided by Simplex Centroid Design (Table 1). Table 1 also shows the effects of different solvents on response variables: TPC, TFC, antioxidant activity (EC_50_, measured by DPPH radical scavenging determination), HEp-2 antiproliferative activity (HEp-2), and mononuclear cell viability (MCV).

### Model fitting: initial considerations

Analysis of variance (ANOVA) was used for the evaluation of the fitted mathematical model, with a confidence interval of 95% (Table 1). ANOVA analyses were done to choose the best model for TPC, TFC, antioxidant activity (measured EC_50_), HEp-2, and MCV. The model performance was determined by calculating the R^2^ coefficient. From the literature, it is usual to consider a mathematical equation reasonable when R^2^ > 70% because this determination coefficient indicates the suitability of the regression model. However, the closer the R^2^ value to unity, the better and more significantly the model fits to the real data [23, 30]. In our work, the R^2^ values for TPC (0.7514, linear model), TFC (0.8272, linear model), antioxidant activity (0.8067, reduced quadratic model), HEp-2 (0.8839, linear model), and MVC (0.8931, reduced special cubic model) indicated that the models were able to explain 75, 83, 81, 88, and 93% of experimental data variability, respectively. Adjusted R^2^ was used to analyze how the experimental results fitted with theoretical results. In our work, adjusted R^2^ ranged from 0.6962–0.9320, which showed that the experimental results fitted with the theoretical results (Table 2).

**Table 2:** ANOVA results for response surface models used subsequently in optimizing TPC, TFC, EC_50_, HEp-2 and MVC on the extraction parameters from *E. punicifolia* leaves

TPC			TFC		EC_50_		HEp-2		MVC	
	Linear model	Lack of fit	Linear model	Lack of fit	Reduced quadratic model	Lack of Fit	Linear model	Lack of fit	Reduced model	Lack of Fit
F	13.60	1.36	23.94	1.60	11.13	1.84	30.46	1.83	23.99	1.40
p	0.0019	0.4179	0.0002	0.3794	0.0032	0.3937	0.0002	0.3941	0.0018	0.4074
R^2^	0.7514		0.8272		0.8067		0.8839		0.9320	
AdjR^2^	0.6962		0.7835		0.7342		0.8549		0.8931	
CV (%)	2.57		4.53		14.64		10.37		6.73	

The coefficient of variation (CV) is a measure of deviation from the mean value, indicating the precision and repeatability of an assay. It is usual to consider CV < 10% to indicate a reliable and precise model. CV values found in this manuscript (Table 2) showed the reliability and precision of the model. These results demonstrated that the variability of the model was compatible with the variability that occurred experimentally.

A regression model obtained from ANOVA analysis was evaluated by using F statistics and a lack of fit test. It was considered a highly significant model if, besides the low value of p (< 0.05), the computed F-value was greater than the tabulated F-value.

From Table 2, ANOVA indicated a significant regression for the linear models for TPC and TFC at the 95% confidence level (TPC: F = 13.60, p = 0.0019; TFC: F = 23.94, p = 0.0002). Additionally, the lack of fit test was not significant for these models (TPC: F = 1.36, p = 0.4179; TFC: F = 1.60, p 0.3794). For EC_50_, the reduced quadratic model was significant (F = 11.13, p 0.0032) and the lack of fit test was not significant (F = 1.84, p 0.3934). A linear model for HEp-2 (F = 30.46, p = 0.0002) and reduced special cubic for MVC (F = 23.99, = 0.0003) were significant, with the lack of fit not significant for either (F = 1.83, p 0.3941; F = 1.40, p 0.4074, respectively).

### Effects of the solvent system on the response variable

#### Total phenol content (TPC) and total flavonoid content (TFC)

In the literature, there is not a general consensus regarding the use of a single solvent or mixture of solvents ideal for obtaining bioactive compounds from plant materials, however it is frequently stated that hydroethanolic extraction is the most appropriate alternative to recover phenolic compounds from plants [31]. Compared to the alternatives, water alone was not a good cold solvent extractor (at room temperature). Nevertheless, the negative effect of its presence in a mixture was not too expressive, since aqueous mixtures had interesting results too. This situation was also described by Kalia et al. [24], where it was defined that a 50% ethanol-aqueous mixture was the most efficient for extracting polyphenols.

Phenolic compounds, such as acids, flavonoids, and tannins, could be differentially influenced by the polarity of the solvents and the solubility of each compound in the solvent used for the extraction process. Methanol, ethanol, water, or mixtures of these solvents are used to extract phenolic compounds from plant parts like leaves, roots, and fruits. Ethanol or ethanol–water mixtures are the most used solvents for extraction because of their low toxicity. Moreover, hydroethanolic solutions have some similarities with popular medicinal Brazilian preparations, usually known as “garrafada”—a preparation made by plant part maceration in brandies like “cachaça” [32]. However, there are some reports that correlate methanol and methanolic solutions to flavonoid enriched extracts [33]. In summary, the solvent choice is always made to get the best phenolic extraction performance with the maximum biological activity possible and with minimum toxicity.

Considering only the significant effects, the linear model is represented by polynomial Eq. 3:

3 $$ \varvec{TPC} = 282.62 w + 339.68e + 327.39m $$

**Fig. 2:**
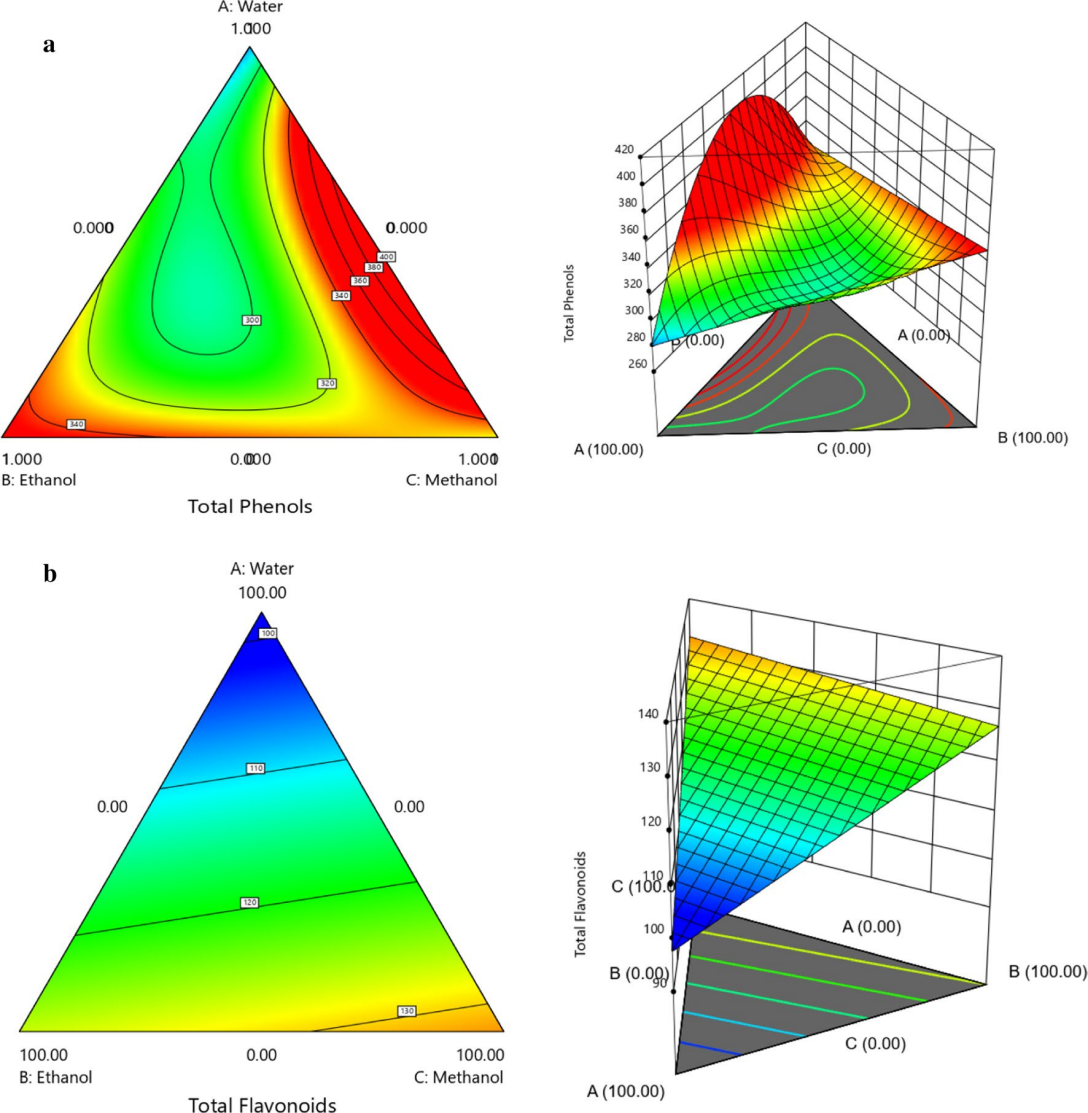
Contour and three-dimensional graph for the analysis of the linear model’s response surface predicted for the extraction of total phenols and flavonoids content according to the percentage of the solvents: **a** water, **b** ethanol, **c** methanol

All linear terms were positive and the highest coefficient value from Eq. 3 (+339.68) showed ethanol as the solvent that enhanced phenolic extraction the most, followed by methanol (+ 327.39) and water (+ 282.62). These solvents therefore had a positive effect on the recovery of phenolic compounds. From Eq. 3, a three-dimensional plot (Fig. 2a) was proposed to get a better picture and interpretation of the adjusted mathematical model, and to further investigate the effects of interactions between the variables studied. Through Fig. 2a, it could also be seen that the ethanolic extraction enabled a higher quantity of phenolic compounds, but when ethanol was at lower concentrations in the mixture, lower quantities of phenolics were obtained. On the other hand, water also demonstrated a positive effect when mixed with ethanol. Confirming this trend, TPC contents oscillated between 164.15 ± 0.02 mg GAE/g extract (MeOH:H_2_O 50%, EE13) and 412.59 ± 0.01 mg GAc/g extract (EtOH 100%, EE5).

Comparing different methods of polyphenol solvent extractions from *Eugenia uniflora* leaves, great variation was observed in the polyphenol content (51–504 mg GAc/g dry extract) and ethanolic extracts also had more expressive results [34]. For EtOH: H_2_O solvent mixtures, *E. punicifolia* produced 74.86 ± 0.02 mg GAc/g from hydroethanolic leaf extracts (EtOH:H_2_O 70%) [7]. The current results proved consistent with the literature since, in the hydroethanolic extracts cited above, phenolic compounds such as gallic, quinic, and syringic acids, and derivatives such as glycosyringic and ferruloylquinic acid, were mainly found [7].

For flavonoids, the best solvent extractor was not one of the extremes of the dielectric constant, because these phenolics present different polarities. This is because phenolic compounds include a broad range of compounds, from simple phenols with low molecular weight and a single aromatic ring to large, complex tannins and derived polyphenols [1].

The TFC linear model is represented by polynomial Eq. 4:

4 $$ \varvec{TFC} = 97.80w + 127.18e + 132.14m $$

The Eq. 4 terms suggested that each component of the mixture generates a positive (synergistic) effect on total flavonoid recovery. Methanol (+ 132.14) was a little more effective than ethanol (+ 127.18), but there was a decrease in the flavonoid content after the introduction of water into the solvent mixture. Therefore, ethanol-enriched solvents would be much more interesting in extraction, due to their low toxicity, but extracts made from methanol were richer in flavonoid content (Fig. 2b).

**Fig. 3:**
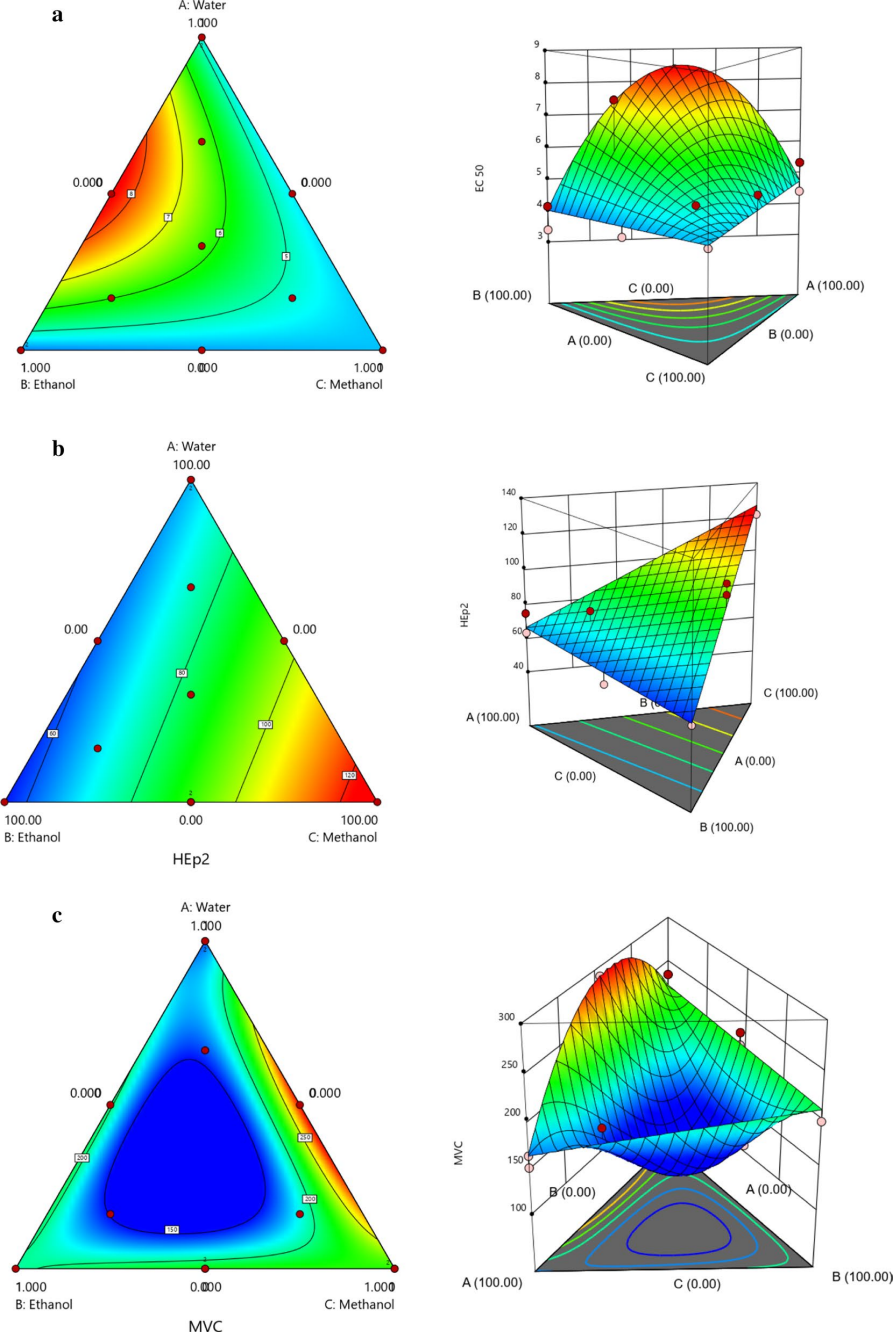
Contour and three-dimensional for the analysis of the model’s response surface predicted for EC_50_ (**a**), HEp-2 (**b**) and mononuclear cell viability (**c**)

Analyzing the data from Table 1 and comparing with Fig. 3, it can be seen (in light red) that 100% MeOH is the best solvent for flavonoid extraction (137.43 ± 0.02 mg Q/g, EE6), followed by EtOH:MeOH 50% v/v (131.32 ± 0.04, EE10). This tendency was observed in Fig. 2b from pure H_2_O (101.86 ± 0.01 mg Q/g, EE4). From our own results, a lower content of these phenolics was produced by *E. punicifolia* leaf extracts using EtOH:H_2_O 70% v/v (32.00 ± 0.02 mg Q/g extract) when compared to the other *Eugenia* spp. [7].

#### Antioxidant activities

An antioxidant is defined as a molecule capable of slowing or preventing the oxidation of other molecules [35]. These molecules prevent mutations to macromolecules by quenching ROS and decreasing the ROS induced oxidative damage. An epigallocatechin gallate from green tea and genistein from soybeans can also act as inhibitors of angiogenesis [36, 37] and DNA methyltransferases (DNMT) in vitro [38]. Accordingly, there are many reviews on the relationships between oxidative damage and various diseases including Alzheimer’s disease, ageing, arthritis, inflammation, diabetes, Parkinson’s disease, and atherosclerosis, among others. As a result, many diseases have been treated with antioxidants to prevent oxidative damage [35].

The DPPH assay is commonly applied to the investigation of antioxidant molecules because it is a simple and sensible method. The DPPH radical acts receiving a hydrogen from an antioxidant molecule, and this change is noticed by a spectrophotometer as the colour is altered from purple to yellow, with the absorbance at 517 nm decreasing proportionally [35]. Therefore, the lower the EC_50_, the more active the compound is. The result is independent of the concentration of the sample and comparison with other extracts is more simple and trustworthy [39].

The positive terms reduced quadratic model for antioxidant activity, measured by EC_50_, suggested that each component of the mixture generated a positive impact on the antiradical effect (Eq. 5). The fluctuation in EC_50_, depending on the different proportion of solvents tested here (water/ethanol/methanol), can also be seen in the Additional file 1: Figure S1.

5 $$ \varvec{EC}_{50} = 4.76w + 4.01e + 4.33m + 16.37 we $$

Since lower values of EC_50_ present better antioxidant activity, Ethanol (+4.01) is a little more effective than methanol (+ 4.33) and water (+ 4.76). Moreover, if water was introduced in the solvent mixture, there was an increase in the EC_50_ value (synergistic effect) and a loss of activity (Fig. 3a). From Table 1, the best antioxidant capacity was found with 100% EtOH (3.39 ± 0.00 μg/mL, EE1), but in general there was an EC_50_ range of 3.39–8.38 µg/mL. This result indicated that leaf extraction generated very active extracts, i.e., all of them had a good capacity for free radical scavenging.

For flavonoids, the antioxidant activity depends of their proton donor ability which always correlated with of the number of hydroxyl groups in these phenolics [10]. For example, quercetin has been greater antioxidant activity if compared a rutin, a quercetin glycosylated molecule, because of the hydroxyl groups available [8]. Thus, quercetin and myricetin derivatives found in *Eugenia* extracts seem to be determinants for antioxidant activity [17]. For hydrolyzable tannins, a correlation between antiradical activity measurements and molecular weight of ellagitannins and ellagic acid was described. Tannins’ antioxidant capacity is also attributed to the presence of hydroxy functions, which exhibit a greater ability to donate a hydrogen atom and support the unpaired electron compared to those of low molecular weight tannins, but the presence of a sugar moiety would decrease this effect [40].

As described here, the ethanolic leaf extracts of *E. pyryformis* presented a yield EC_50_ of 1.10 µg/mL. However, using the same extract conditions, *E. chlorophylla* showed an EC_50_ of 33.72 µg/mL [41] and *Myrcia* spp. (Myrtaceae) reached similar results to DPPH (EC_50_ = 8.61–16.2 µg/mL) from EtOH:H_2_O 70:30% extracts [19]. For this reason, it is always good to keep in mind that it is necessary to do a new test for each plant, since the chemical composition could be different.

#### Antiproliferative activities: HEp-2 and MCV

Antiproliferative activity is the first step in anti-cancer chemotherapeutic agent analysis. Many studies have demonstrated that polyphenols, including flavonoids, can act through several mechanisms of action at different points during the carcinogenesis process, either alone or in a synergistic way [42]. The positive terms for the linear model tested for antiproliferative against Hep-2 cells are represented by polynomial Eq. 6:

6 $$ \varvec{HEp}2 = 66.39w + 55.76e + 127.11m $$

From the above equation, the antiproliferative activity against HEp-2 from ethanol (+ 56.76) and water (+ 66.39) could be established, whilst extracts prepared from methanol (+ 127.11) seemed to have discrete proliferative activity, as this term exceeded 100%. This trend could be better observed from Fig. 3b, where methanol extracts showed the higher response. Since methanol rich extracts were flavonoid rich, it was expected that those phenolics were responsible for the observed effect.

From Table 1, moderate antiproliferative activity against HEp-2 could be observed at first glance from EE6 (43.37 ± 0.19%, 100% MeOH) and EE5 (54.60 ± 0.17%, 100% EtOH). However, for the replicate extracts EE14 (121.87 ± 0.04%, 100% MeOH) and EE2 (189.90 ± 0.33%, 100% EtOH), the opposite effect was observed, i.e., proliferative activity. Thus, the simplex centroid design was crucial in determining the effect of the extracts on the analyzed response.

The next step was describing the effects of solvent extraction on mononuclear cell viability. The fluctuation in cell viability results, depending on the different proportion of solvents tested here (water/ethanol/methanol), can also be seen in the Additional file 1: Figure S1. In Eq. 7, a synergistic trend of MeOH:H_2_O (+ 395.29) and MeOH (+ 223.57) extracts increasing the cell viability was noted. The most interesting result was the antagonistic effect exerted by the ternary mixture (− 3976.89). This trend, confirmed by data from Table 1 and Fig. 3c, showed a high value for EE13 (290.06 ± 13.60, MeOH:H_2_O 50:50 v/v) followed by EE14 (236.67 ± 20.37, 100% MeOH).

7 $$ \varvec{MCV} = 159.15w + 196.29e + 223.57m + 110.05 we + 395.29wm + 40.04  em - 3976.89  wem $$

There is a coherence between the results above, since the same extracts show proliferative activity and growth of normal cells. These results were interpreted as an effect of flavonoid enriched extracts. Myrtaceae plants are described as rich in flavanol derivatives such as quercetin and myricetin [19]. Quercetin is usually described as being able to react with ROS, both in vitro and in vivo. This molecule acts as an anti-tumour drug in vitro by exerting pro-apoptotic activity, especially on leukemic cells, but not on immune cells, as several chemotherapeutic drugs do [43].

Myricetin acts as an antioxidant at lower concentrations and it has pro-oxidant effects at higher concentrations. The presence of the catechol moiety in the B-ring was linked to a strong DPPH scavenging activity. Myricetin is also described as cytotoxic towards several human cancer cell lines, including hepatic, skin, pancreatic, and colon cancer cells. When there is an increase in the concentration, this molecule showed an enhanced anti-proliferative activity against HL-60 cells. This compound displayed cytotoxicity towards chronic myeloid human leukaemia K562 cells and normal peripheral blood mononuclear cells isolated from the blood of a healthy humans, and it also exhibited moderate cytotoxicity towards human laryngeal carcinoma HEp-2 cells [17].

Unfortunately, less information is available regarding possible synergistic or antagonistic biochemical interactions among polyphenols that may affected their antiproliferative activity. Identifying potential interactions among these compounds may help define the efficiency of polyphenol-containing extracts in cancer prevention and relate this to the structure function activity of the compounds. For example, quercetin and ellagic acid were tested on cell death and proliferation-related variables. In this case, ellagic acid had potentiated the effects of quercetin on the reduction of proliferation and viability and the induction of apoptosis in the MOLT-4 human leukaemia cell line [44].

Mononuclear cell proliferation may indicate lymphocyte proliferation, which is responsible for the memory of our immunity [45]. It would be ideal if one of the tested extracts had both functions, i.e., increasing mononuclear cell proliferation and reducing tumour cell proliferation, but any reduction in tumour cell proliferation should be considered a good result because it helps in reducing tumour mass.

### ESI–MS analysis

The chemical profile of the *E. punicifolia* extracts, determined by ESI(−)-Q/TOFMS, allowed for identification of phenolic acids, quercetin and myricitrin derivatives, and other phenolic compounds. The most characteristic corresponding molecular formulas and their fractions and MS/MS fragments are shown in Table 3.

**Table 3:** Phenolic identified by ESI(−)-MS found in ethanolic Eugenia punicifolia extracts

Formula [M−H]^−^	Theoretical mass [M−H] *m/z*	Experimental mass [M−H]^−^ *m/z*	Δm (ppm)	MS/MS fragments *m/z*	Compound identification
C_14_H_5_O_8_	300.9990	300.9990	− 0.03	283, 271, 245, 201, 145, 123	Ellagic acid
C_13_H_15_O_10_	331.0671	331.0666	1.42	271, 211, 169	Monogalloylglucose
C_20_H_17_O_11_	433.0776	433.0767	2.15	300, 271, 151,	Quercetin-3-*O*-β-d-xylopyranoside
C_21_H_19_O_11_	447.0933	447.0940	− 1.60	316, 300, 271, 255, 169	Quercetin-3-*O*-β-rhamnoside
C_18_H_23_O_14_	463.0891	463.0882	− 1.92	316, 271, 169, 125	Myricitrin
C_20_H_17_O_14_	481.0624	481.0642	2.86	301, 275, 169, 151	HHDP glucose isomer
C_20_H_19_O_14_	483.0780	483.0756	5.02	331, 169, 125	Digalloyl-glucose isomer
C_39_H_18_O_8_	615.1085	615.1061	3.96	463, 300, 169	Quercertin galloylhexoside isomer
C_34_H_23_O_22_	783.0686	783.0678	1.08	617, 300, 275	Bis HHDP-glucose isomer

**Fig. 4:**
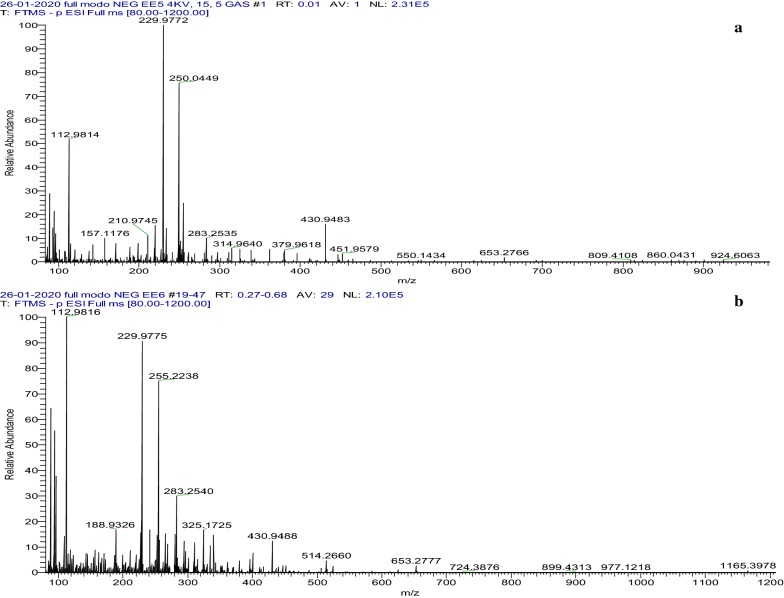
ESI(−)-MS of the selected *E. punifolia* leaves extract. **a** Ethanolic extract (EE5). **b** Methanolic extract

All peaks were tentatively assigned based on their accurate masses and MS/MS patterns. Monogalloylglucose, with its m/z 331 [M–H]^−^ ion, dissociated to yield an m/z 169 ion after a glucosyl group loss [M−H-162]^−^. Digalloylglucose, with its m/z 483 [M−H]-ion, dissociated to yield an m/z 169 ion after sequential removal of a galloyl group [M−H-152]^−^ and a glucosyl group [M−H-162]^−^. The quercetin arabinopyranoside isomer at m/z 433 [M−H]^−^ produced the MS/MS fragmentation of m/z 300 [M−H-132]^−^ due to the loss of pentoside. The ion at m/z 447, assigned to quercetin-3-*O*-β-rhamnose, after a loss of sugar, produced a deprotonated aglycone ion at m/z 301 [M−146-H,]^−^. Myricitrin, was assigned at m/z 463 [M–H]^−^, after a deprotonated a myricetin ion at m/z 317 [M−146-H]^−^. At m/z 615, the MS/MS peak fragmentation produced an ion at m/z 463 [M−152-H] ^−^ and a deprotonated quercetin at m/z 301 [M−162-H]^−^, indicative of quercetin-3-*O*-β-(6″galloyl) hexose. These flavanol derivatives were previously reported in other Eugenia species and are usually associated with antioxidant and antiproliferative activities. Ellagic acid was characterized by an ion of m/z 301[M-162-H]. HHDP-glucose isomers were assigned as a signal at m/z 481[M-162–18-H] (loss of glucose plus H_2_O, 180 units). At m/z 783, the MS/MS peak fragmentation produced an ion at m/z 481 [M−H-302]^−^ and after losing an HHDP-glucose [M−H-481]^−^, an ion at m/z 301 which assigned to ellagic acid. This fragmentation pattern was assigned to a bis-HHDP-glucose isomer [19]. All these results are consistent with data reported for other Eugenia spp. [7, 19] (Fig. 4).

## Conclusion

Since the purpose of this work is to report the optimized studies of solvent extraction that result in a better biological activity, it is possible to point out some inferences. From simplex centroid design analysis, the extraction of antioxidant compounds from *E. punicifolia* leaves showed that solvents richer in ethanol were the most adequate solution. Since this solvent is the best extractive for all kind of phenolic compounds, this activity was linked to compounds such as phenolic acid and tannins. This solvent, less toxic than methanol, still has a small antiproliferative activity against HEp-2 and some mononuclear cell viability. These results encourage us in the search for more industrial applications of this extract since they are very promising.

## Supplementary information

**Additional file 1: Figure S1.** DPPH antiradicalar effects. Cell viability from Hep-2 and mononuclear cells incubated with different extracts. The numbers below each column is correspondent those Table 1. The difference statistical (p < 0.05) between the tested extracts is appointed by numbers above each column.

## Data Availability

The datasets used and/or analysed during the current study are available from the corresponding author on reasonable request.

## Abbreviations

DPPH: 2,2-diphenyl-1-picrylhydrazyl
EC_50_: Half maximal effective concentration
ESI-QTOF-MS: Electrospray Ionization Quadrupole time-of-flight Mass Spectrometry
HEp-2: Human epithelial type 2
HHDP: Hexahydroxydiphenoyl
ROS: Reactive oxygen species
