# Supplementary material for: Optimization of Eugenia punicifolia (Kunth) D. C. leaf extraction using a simplex centroid design focused on extracting phenolics with antioxidant and antiproliferative activities
Source: BMC Chem. 2020 Apr 27;14(1):34. doi: 10.1186/s13065-020-00686-2 (PMC7187488; doi:10.1186/s13065-020-00686-2)
Supplement: Supplementary file 1 — Additional file 1: Figure S1. DPPH antiradicalar effects. Cell viability from Hep-2 and mononuclear cells incubated with different extracts. The numbers below each column is correspondent those Table 1. The difference statistical (p < 0.05) between the tested extracts is appointed by numbers above each column. [file 13065_2020_686_MOESM1_ESM.docx]

Additional material. DPPH antiradicalar effects.Cell viability from Hep-2 and mononuclear cells incubated with different extracts. The numbers below each column is correspondent those Table 1. The difference statistical (p<0.05) between the tested extracts is appointed by numbers above each column.
